# Association of a novel homozygous mutation in the *HMGCS2* gene with an HMGCSD in an Iranian patient

**DOI:** 10.1002/mgg3.1507

**Published:** 2020-09-23

**Authors:** Masoud Heidari, Morteza Soleyman‐Nejad, Alireza Isazadeh, Javad Shapouri, Mohammad Hossein Taskhiri, Roghayyeh Ahangari, Ali Reza Mohamadi, Masoumeh Ebrahimi, Hadi Karimi, Manzar Bolhassani, Zahra Karimi, Mansour Heidari

**Affiliations:** ^1^ Department of Animal Biology Faculty of Natural Sciences University of Tabriz Tabriz Iran; ^2^ Ariagene Medical Genetics Laboratory Qom Iran; ^3^ Immunology Research Center Tabriz University of Medical Sciences Tabriz Iran; ^4^ Pediatric Clinical Research and Development Center Qom University of Medical Sciences Qom Iran; ^5^ Department of Cellular and Molecular Genetics Islamic Azad University, Qom Branch Qom Iran; ^6^ Department of Obstetrics and Gynecology Nekouei‐Hedayati‐Forghani Hospital Qom University of Medical Sciences Qom Iran; ^7^ Qom Social Welfare and Rehabilitation Center Qom Iran; ^8^ Department of Medical Genetics Tehran University of Medical Sciences (TUMS Tehran Iran

**Keywords:** *HMGCS2* gene, HMGCSD, metabolic disorder, Sanger sequencing, whole‐exome sequencing

## Abstract

**Background:**

3‐Hydroxy‐3‐methylglutaryl‐CoA (HMG‐CoA) synthase 2 gene (*HMGCS2*) encodes a mitochondrial enzyme catalyzing the first reaction of ketogenesis metabolic pathway which provides lipid‐derived energy for various organs during times of carbohydrate deprivation, such as fasting. Mutations in this gene are responsible for HMG‐CoA synthase deficiency (HMGCSD). The aim of present study was to investigate the association of mutation in the *HMGCS2* gene with HMGCSD in a patient with atypical symptoms.

**Methods:**

The clinical and genetic features of an 8‐months‐old girl with HMGCSD were evaluated. Molecular genetic testing was conducted using whole‐exome sequencing (WES) in order to identify potential disease‐causing mutation. The WES finding was confirmed by the polymerase chain reaction (PCR) amplification of the target sequence carried out for the patient and her parents. The PCR products were subjected to direct sequencing using forward and reverse specific primers corresponding to the *HMGCS2* gene.

**Results:**

A novel homozygous missense mutation (c.266G>A p.Gly89Asp) was detected in the *HMGCS2* gene. Sanger sequencing along with co‐segregation analysis of all family members confirmed this novel pathogenic germline mutation. The mutant gene was found to be pathogenic by bioinformatics analysis.

**Conclusion:**

To our best knowledge, this is the first report of HMGCSD in Iran which would expand our knowledge about the mutational spectrum of the *HMGCS2* gene and the phenotype variations of the disease.

## Graphical abstract

The HMGCS2 enzyme catalyzing first reaction of ketogenesis and mutations in this gene are responsible for HMGCSD. Mutations of the HMGCS2 gene was evaluated in a girl (8 months old) with HMGCSD using WES method. We found a novel homozygous missense mutation (c.266G>A p.Gly89Asp) in the HMGCS2 gene and approved by Sanger sequencing.

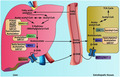

## 1 INTRODUCTION

The Ketone bodies are produced from fatty acids and provide an alternative energy source in times of glucose deficiency (Sass, 2012). The mitochondrial 3‐hydroxy‐3‐methylglutaryl‐CoA (HMG‐CoA) synthase enzyme is involved in catalyzing condensation reaction between acetoacetyl‐CoA and acetyl‐CoA to form HMG‐CoA, which is the rate‐limiting step of ketone bodies synthesis (Hegardt, 1999).

**FIGURE 1:**
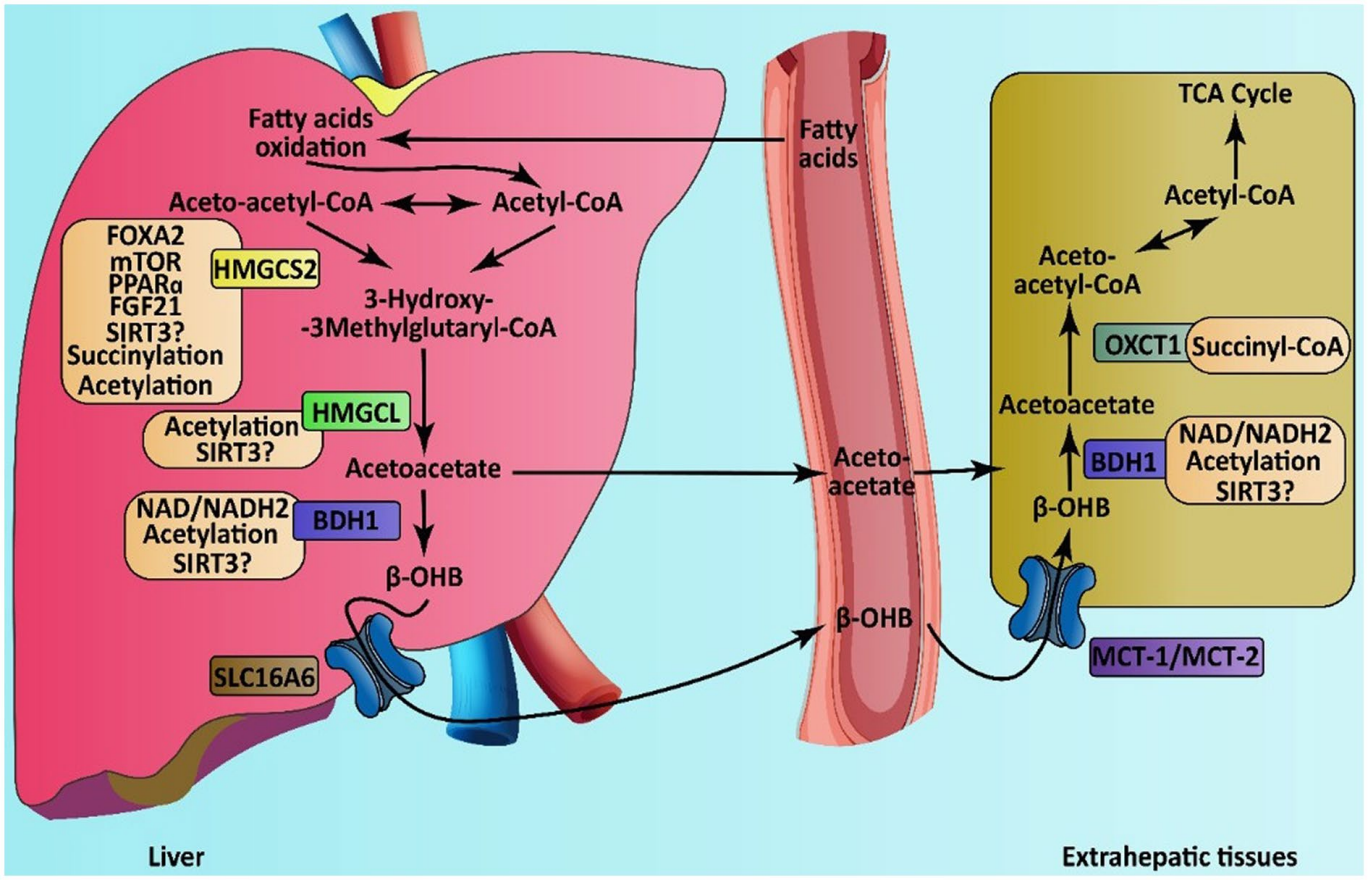
Regulation and metabolism of ketone body. The synthesis of 3‐hydroxy‐3‐methylglutaryl‐CoA by *HMGCS2* is the key step in ketogenesis. The transcription of the *HMGCS2* gene regulated by FGF21, PPARα, mTOR, and FOXA2. The *HMGCS2* activity regulated by succinylation, acetylation, and SIRT3 deacetylation

The 3‐hydroxy‐3‐methylglutaryl‐CoA lyase deficiency (HMGCLD) is a rare autosomal recessive inborn error of ketone body synthesis and leucine degradation caused by the mutant forms of HMG‐CoA synthase 2 (*HMGCS2*) gene (OMIM: 600234) (Figure 1). This gene encodes a mitochondrial enzyme that controls the HMG‐CoA cycle, by which acetoacetate, β‐hydroxybutyrate, and NAD^+^ are produced (Shimazu et al., 2010).

The first HMGCLD case was reported by Thompsom et al. in 1997 (Thompson, Hsu, Pitt, Treacy, & Stanley, 1997). So far, more than 20 cases have been reported throughout the world among different ethnic groups (Pitt et al., 2015; Puisac et al., 2018; Ramos et al., 2013). These patients manifest hepatomegaly and hypoglycemia during acute infection and prolonged fasting. The elevated plasma levels of free fatty acids and non‐(hypo)‐ketotic hypoglycemia are additional important findings (Fukao et al., 2014). The clinical presentations of mitochondrial HMG‐CoA synthase (mHS) deficiency are similar to the defects in the fatty acid β‐oxidation pathway. While the evaluation of 4‐hydroxy‐6‐methyl‐2‐pyrone (4HMP) in urine is used as a diagnostic test for mHS deficiency, however, its diagnosis can often be challenging. Therefore, genetic analysis can provide an important alternative diagnostic method for mHS deficiency detection (Aledo et al., 2006).

In the present study, we report the first Iranian HMGCSD case. The patient is an 8‐month‐old Iranian girl who presented to the hospital predominantly with repeated vomiting and severe loss of consciousness as well as with normoglycemia at the acute phase. The patient underwent a comprehensive clinical and paraclinical examination. In addition, we characterized a novel homozygous mutation in the *HGMCS2* gene by whole‐exome sequencing.

## 2 METHODS AND MATERIALS

### 2.1 Case presentation

**FIGURE 2:**
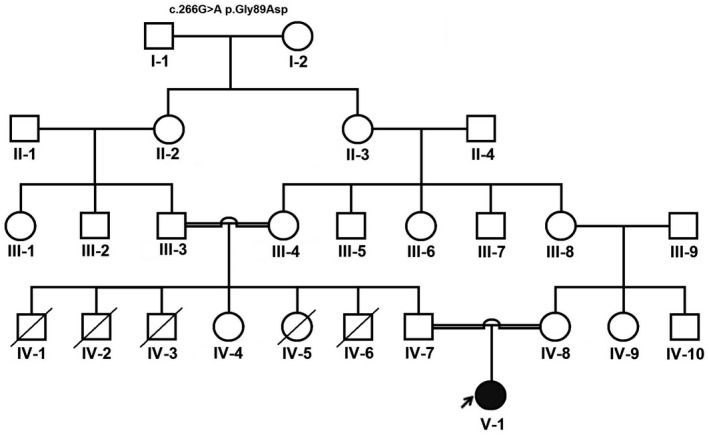
The pedigree analysis of a patient with *HMGCS2* gene mutation. The arrow indicates the proband

An 8‐month‐old Iranian girl referred to Hazrat Masoumeh Children Hospital and Training Center (Qom, Iran) in April 2019 after 3 days with upper respiratory symptoms, frequent fever, and vomiting, tachypnea, generalized tonic‐clonic seizure, loss of oral intake, and decreased consciousness. She was the first child born to a consanguineous couple, without any physical and mental growth abnormalities (Figure 2). The pedigree designed was performed using the Cyrillic software. Following the Declaration of Helsinki ethical standards, parents were informed about the study and signed informed consent.

### 2.2 Whole‐exome sequencing (WES)

The peripheral blood samples (3 ml) were obtained from the proband and her parents. The extraction of genomic DNA was conducted by DNA purification kit, according to the manufacturer's instructions. The quantity and quality of extracted genomic DNA were evaluated using electrophoresis on 1% agarose gel and nanodrop instrument. The whole‐exome sequencing method was used to enrich genomic coding regions and other important genomic regions. Also, the next‐generation sequencing (NGS) method was used to sequence ~100 million reads on Illumina Sequencer (Illumina, San Diego, CA, USA) which provided a platform to examine >95% of targeted regions with a sensitivity of above 99%, and to detect point mutations, micro‐insertion/deletions, and duplications (<20 bp). The sequencing results were analyzed using international databases. The SAMtools and ANNOVAR software packages were used for the identification and analysis of genetic variants, such as point mutations and indels. A 45 candidate gene was considered a variant that fulfilled the following criteria: 1: frameshift, nonsense, splice site variants, and missense; 2: absent or rare (frequency below 1%) in the two databases (dbSNP, 1000G); 3: homozygous variants in the patient (Heidari, Soleyman‐Nejad, et al., 2019; Heidari, Soleyman‐Nejad, Isazadeh, et al., 2020).

### 2.3 Sanger sequencing

The fragment of the *HMGCS2* gene with target mutation was amplified using 10 pmol of each forward primer TGCTATGCAGCCTACCGCAAGA and reverse primer GCCAGGGATTTCTGGACCATCT, DNA polymerase (0.2 U Taq), dNTPs (200 μM), MgCl_2_ (0.5 mM), template DNA (60 ng), and PCR buffer in 25 μl total volume of PCR reaction. The PCR conditions include the initial denaturation step (1 cycle for 3 min at 95°C), denaturation step (35 cycles for 30 sec at 95°C), annealing step (35 cycles for 30 sec at 60°C), extension step (35 cycles for 30 sec at 60°C), and final extension (1 cycle for 10 min at 72°C). The amplified fragment was separated on 2% agarose gel and stained by Green Viewer. To confirmation of the target mutation, the PCR products were subjected to direct sequencing. The Sanger sequencing was performed using ABI 3130 automated sequencer (Applied Biosystems, Forster City, CA, USA). The sequencing data were searched in non‐redundant nucleic and protein databases BLAST (http://www.ncbi.nlm.nih.gov/BLAST) (Ahmadi et al., 2018; Heidari, Soleyman‐Neja, et al., 2019).

## 3 RESULTS

### 3.1 Clinical laboratory finding

Clinical laboratory tests showed mild hyperammonemia (133 μmol/L), elevated aspartate, and alanine aminotransferases and alkaline phosphatase (SGOT 172 IU/L, SGPT 86 IU/L, and ALP 687 IU/L), elevated alanine aminotransferase (ALT 161 IU/L), severe metabolic acidosis (pH 7.08, pO2 115 mmHg, and HCO_3_ 2.3 mmol/L), and hypoglycemia. The pyruvic acid, lactic acid, serum creatine phosphokinase (CPK) level, and plasma acyl‐carnitine profile were normal. Plasma amino acids profile obtained by high‐performance liquid chromatography (HPLC) was within the normal range for age. Increased glutaric acid (21.8 mmol/mol, normal: ˂7) was detected in urine organic acid testing. The observed signs treated with infusing dextrose 10% along with 100 meq/L of bicarbonate 8.4%, carnitine, and vitamin cocktail (vitamin B1, B2, B12, C, and B6) led to the rapid recovery of acidosis and consciousness defect. However, after a few weeks, the patient returned to the hospital with vomiting, mild metabolic acidosis, and upper respiratory tract viral infection symptoms, although the level of consciousness was intact. In clinical examination, no other abnormal finding was observed other than the depressed level of consciousness, Kussmaul breathing, and marked hepatomegaly.

### 3.2 Genetic analysis finding

Whole‐exome sequencing revealed a novel homozygous missense mutation (c.266G>A p.Gly89Asp) in the *HMGCS2* gene. This variant was confirmed by PCR direct sequencing. The pathogenicity of the identified mutation was studied using co‐segregation analysis on all family members as well as by the bioinformatics study. The results confirmed the diagnosis of mHS deficiency.

## 4 DISCUSSION

Deficiency of mitochondrial HMG‐CoA synthase (mHS) is a metabolic disorder with autosomal recessive inheritance, which impacts the synthesis of ketone bodies. The important complications include dicarboxylic aciduria, hepatomegaly, lethargy, hypoglycemia, vomiting, and coma (Aledo et al., 2001). The mHS deficiency has been under‐diagnosed owing to the absence of specific biochemical and clinical markers, liver biopsy limitations, and the lack of a method of enzyme assay for verifying underlying mutations (Zschocke et al., 2002). Hence, the molecular analysis can be an effective method for the diagnosis of this disorder. However, molecular analysis has been limited to measure the activity of the cloned mHS enzyme (Shafqat, Turnbull, Zschocke, Oppermann, & Yue, 2010).

In the present study, we reported a patient with mHS deficiency, which was the first diagnosed case in Iran. Like the other reported patients with mHS deficiency, our proband presented with severe metabolic acidosis, fatty liver, severe fatty liver, hepatomegaly during poor intake, and acute infection. We screened the whole *HMGCS2* gene by NGS technology in the studied patient and discovered a novel *HMGCS2* missense mutation (c.266G>A p. Gly89Asp) causing mHS deficiency. Moreover, the in silico analysis (position of amino acid change, nature, and evolutionary conservation) showed that the identified mutation is able to damage the function and structure of the *HMGCS2* coding protein.

In this family, the proband with mHS deficiency was demonstrated to harbor a novel *HMGCS2* homozygous substitution mutation, that is, c.266G>A p. Gly89Asp. This mutation, located in exon 2, is an mHS pathogenic mutation that damages the translation of the protein. The affected proband in our study manifested mHS deficiency phenotypes which were similar to those observed in previous cases (Bouchard et al., 2001; Wolf, Rahman, Clayton, & Zschocke, 2003). The clinical evaluation in the present study demonstrated an apparent genotype‐phenotype association in this disorder. In a study by Ramos et al. (2013), reported eight mutations, which four of them not produced the proteins, third mutations were loss of activity, and one mutants were enzyme catalytic efficiency (Ramos et al., 2013).

## 5 CONCLUSION

In conclusion, our study characterizing a novel mutation in *HMGCS2* gene extending the mutational spectrum in HMGCSD. However, further studies are required to clarify genotype‐phenotype association in patients with various mutations on the *HMGCS2* gene.

## CONFLICT OF INTEREST

The authors declare that there is no conflict of interest.

## AUTHOR'S CONTRIBUTION

All authors had an equal role in the design, work, statistical analysis, and manuscript writing.
